# Reference Ranges for NT-proBNP (N-Terminal Pro-B-Type Natriuretic Peptide) and Risk Factors for Higher NT-proBNP Concentrations in a Large General Population Cohort

**DOI:** 10.1161/CIRCHEARTFAILURE.121.009427

**Published:** 2022-09-13

**Authors:** Paul Welsh, Ross T. Campbell, Leanne Mooney, Dorien M. Kimenai, Caroline Hayward, Archie Campbell, David Porteous, Nicholas L. Mills, Ninian N. Lang, Mark C. Petrie, James L. Januzzi, John J.V. McMurray, Naveed Sattar

**Affiliations:** School of Cardiovascular and Metabolic Health, University of Glasgow, United Kingdom (P.W., R.T.C., L.M., N.N.L., M.C.P., J.J.V.M., N.S.).; British Heart Foundation Centre for Cardiovascular Science (D.M.K., N.L.M.), University of Edinburgh, United Kingdom.; Medical Research Council Human Genetics Unit (C.H.), University of Edinburgh, United Kingdom.; Institute of Genetics and Cancer (A.C., D.P.), University of Edinburgh, United Kingdom.; Usher Institute (N.L.M.), University of Edinburgh, United Kingdom.; Cardiology Division, Massachusetts General Hospital, Boston (J.L.J.).; Harvard Medical School, Boston, MA (J.L.J.).; Baim Institute for Clinical Research, Boston, MA (J.L.J.).

**Keywords:** age distribution, demography, heart failure, risk factors, sex

## Abstract

Demographic differences in expected NT-proBNP (N-terminal pro-B-type natriuretic peptide) concentration are not well established. We aimed to establish reference ranges for NT-proBNP and explore the determinants of moderately elevated NT-proBNP under the universal definition of heart failure criteria.

**Methods:**

This is a cross-sectional study. NT-proBNP was measured in serum from 18 356 individuals without previous cardiovascular disease in the Generation Scotland Scottish Family Health Study. Age- and sex-stratified medians and 97.5th centiles were generated. Sex stratified risk factors for moderately elevated NT-proBNP (≥125 pg/mL) were investigated.

**Results:**

In males, median (97.5th centile) NT-proBNP concentration at age <30 years was 21 (104) pg/mL, rising to 38 (195) pg/ml at 50 to 59 years, and 281 (6792) pg/mL at ≥80 years. In females, median NT-proBNP at age <30 years was 51 (196) pg/mL, 66 (299) pg/mL at 50 to 59 years, and 240 (2704) pg/mL at ≥80 years. At age <30 years, 9.8% of females and 1.4% of males had elevated NT-proBNP, rising to 76.5% and 81.0%, respectively, at age ≥80 years. After adjusting for risk factors, an NT-proBNP ≥125 pg/mL was more common in females than males (OR, 9.48 [95% CI, 5.60–16.1]). Older age and smoking were more strongly associated with elevated NT-proBNP in males than in females (*P*_sex interaction_ <0.001, 0.07, respectively). Diabetes was inversely associated with odds of elevated NT-proBNP in females only (*P*_sex interaction_=0.007).

**Conclusions:**

An NT-proBNP ≥125 pg/mL is common in females without classical cardiovascular risk factors as well as older people. If NT-proBNP becomes widely used for screening in the general population, interpretation of NT-proBNP levels will require that age and sex-specific thresholds are used to identify patients with potential pathophysiology.

**What is New?**

The Universal definition of heart failure states that NT-proBNP (N-terminal pro-B-type natriuretic peptide) ≥125 pg/mL is corroborating evidence of heart failure in the acute setting, but this single threshold may not be optimal for screening in the general population. In this general population study, we report that NT-proBNP ≥125 pg/mL is frequently observed in many demographic groups, including in around 10% of young females, many of whom are unlikely to have preclinical heart failure. Indeed, NT-proBNP ≥125pg/mL was much more common in females than in males (adjusted odds ratio 9.48).

**What are the Clinical Implications?**

Clinical indications for NT-proBNP measurement are likely to expand in the general population in the future. The reference ranges we present in this work will help guide understanding of expected NT-proBNP concentrations in the general population, as well as determinants of moderately elevated NT-proBNP.

Before NT-proBNP can be used as a general population screening tool, a better understanding of the definition, determinants, and consequences of moderately elevated NT-proBNP is required. This includes study of younger people in the general population, as well as individuals in middle-age and older-age.

Measurement of BNP (B-type natriuretic peptide) or NT-proBNP (N-terminal pro-B-type natriuretic peptide) is now a cornerstone of many clinical guidelines in the diagnosis of heart failure. The recent universal definition of heart failure position paper specifies an ambulatory NT-proBNP at 125 pg/mL as a corroborating definition of heart failure when accompanied by symptoms or signs of the disease.^1^ Recently updated guidelines from the European Society of Cardiology also specify the rule out heart failure thresholds of NT-proBNP at 125 pg/mL in the nonacute setting,^2^ a threshold which is also supported by a position paper on the use of natriuretic peptides.^3^ The UK National Institute for Health and Care Excellence guidelines specify a rule-out threshold for chronic heart failure at NT-proBNP of 400 pg/mL, with guidance to refer for echocardiography above this level.^4^ The higher threshold in the National Institute for Health and Care Excellence model was chosen due to an original cost-effectiveness analysis (based on data from a diagnostic accuracy study^5^), conducted for the guidelines.

As well as the heart failure diagnosis pathway, there is also increasing interest in using NT-proBNP in a wider range of indications and patient populations, including to predict cardiovascular disease risk in those without established heart failure,^6–8^ to screen for heart failure in the general population as part of an integrated cardiovascular disease risk screening approach,^9,10^ and to use NT-proBNP in emerging conditions such as prognosis of COVID-19 infection.^11^ In each of these settings, higher NT-proBNP concentrations predict future heart failure onset or its complications; however, when using lower NT-proBNP cut-points (such as 125 pg/mL), it is reasonable to expect a significant overlap between those with unsuspected structural heart disease and those without obvious prevalent disease. To incorporate more widespread NT-proBNP testing into clinical practice, it is important to understand what NT-proBNP levels are typically seen in the general population across a wide range of ages. Although consensus has developed around use of an NT-proBNP cut-point of 125 pg/mL as a moderate elevation that may be clinically significant, more understanding is needed regarding factors influencing the biomarker among individuals without established cardiovascular disease. Published data from general populations exploring the age- and sex-stratified reference ranges for NT-proBNP, and these have generally been from smaller cohorts with restricted age ranges, limiting exploration of reference ranges by age and sex stratified groups.^12–14^

The aims of this study were therefore to use a large general population with a wide age distribution to (1) report normative reference ranges for NT-proBNP, (2) report prevalence of participants in different demographic groups with elevated NT-proBNP according to the existing clinical thresholds, and (3) report risk factors associated with elevated NT-proBNP, stratified by sex. We hypothesised that a single threshold to identify moderately elevated NT-proBNP (≥125 pg/mL) in the context of a general population is unlikely to identify participants in all demographic groups with NT-proBNP concentrations that may require clinical investigation.

## Methods

The data, analytic methods, and study materials will be made available to other researchers for purposes of reproducing the results or replicating the procedure subject to a successful project application to the Generation Scotland Access Committee.

### Generation Scotland Scottish Family Health Study (GS:SFHS)

This is a cross-sectional study within the cohort. The recruitment and design of the GS:SFHS has been reported in detail previously.^15,16^ During 2006 to 2010, potential participants (aged 35–65 years) were identified at random from collaborating general medical practices in Scotland and invited to participate. Participants were also asked to identify ≥1 first-degree relative aged ≥18 years who would be able to participate. A total of 21 476 participants aged between 18 and 98 years attended a research clinic in different urban areas of Scotland. At the clinic, participants had physical and clinical characteristics (including systolic blood pressure [SBP] and body mass index [BMI]) measured according to a standardised protocol and had a questionnaire administered (https://www.ed.ac.uk/generation-scotland/for-researchers/generation-scotland). Ethical approval for the protocol and written study materials were received from NHS Tayside Research Ethics Committee (REC reference number 05/S1401/89). All participants gave informed consent.

Past medical history, including a diagnosis of diabetes (type 1 or type 2) and heart disease or stroke, was recorded using a self-reported questionnaire. Scottish Index of Multiple Deprivation (SIMD) scores are national composite measures of socioeconomic deprivation and were derived from participant postcodes, with higher scores indicating greater socioeconomic deprivation.^17^

Fasting blood samples were taken, according to a standard operating procedure, and serum samples were separated. Biochemistry measures including total cholesterol, HDL (high-density lipoprotein) cholesterol, and creatinine was measured at the time of collection and additional serum aliquots were stored at −80 °C for future biochemical analyses. High sensitivity cTnI (cardiac troponin I) and high sensitivity cTnT (cardiac troponin T) were measured as previously described.^18^

### Previous Cardiovascular Disease

Classification of previous cardiovascular disease (including heart failure) at recruitment was based on linked data from the Scottish Morbidity Record (SMR01), and self-reported heart disease or stroke. SMR01 data were used to identify patients who had been hospitalized for coronary heart disease, heart failure, or ischemic stroke at any time before their baseline assessment (using *International Classification of Disease (ICD)-10* codes I20-I25, I50, I42.0, I42.6, I42.7, I42.9, I11.0, I63, I64, G45). Any participant who had self-reported heart disease or stroke or hospitalized with one of these SMR01 diagnostic codes before their study assessment was excluded.

### Measurement of NT-proBNP

NT-proBNP was measured on a cobas e411 analyser (Roche Diagnostics, Basel, Switzerland). The assay was calibrated and quality controlled using the manufacturer’s reagents. Coefficients of variation for NT-proBNP were 4.5 % for the low control and 2.7% for the high control. The study laboratory participated in the National External Quality Assurance Scheme (https://ukneqas.org.uk/) for NT-proBNP during the conduct of the study. The limit of detection of the NT-proBNP assay is set to 10 pg/mL by the manufacturer, and in the current study anything less than the limit of detection was reported as 5 pg/mL for continuous analysis. NT-proBNP measurements were undertaken during a single (second) thaw of stored serum aliquots.

### Statistical Analysis

Participants with missing data for NT-proBNP, previous cardiovascular disease, or with no time of baseline appointment available were excluded from analyses. The sex-stratified GS:SFHS NT-proBNP medians, 95th, 97.5th, and 99th centiles along with associated bias-corrected 90% CIs (percentile 90% CIs for the ≥80 years age band) around the estimates were determined by bootstrapping 5000 samples in each age and sex-specific strata. Quantile regression using fractional polynomials was used to further model the relationship between age (and time of study appointment) with the median and 97.5th centile of NT-proBNP.

The observed prevalence of moderately elevated NT-proBNP above the existing clinical rule out thresholds were explored, in age and sex strata of the population, using 125 pg/mL based on the universal definition of heart failure (and the European Society of Cardiology clinical guidelines), as well as 400 pg/mL based on National Institute for Health and Care Excellence guidelines.^1,4^ The proportion of participants, above existing rule out thresholds was estimated using single sample proportions and 95% CI from binomial exact estimates. Patterns of the prevalence of sociodemographic and classical cardiovascular risk factors across the distribution of NT-proBNP were explored by splitting NT-proBNP into approximate quintiles (not sex-specific), reserving the top quintile for participants with NT-proBNP above the 125 pg/mL threshold. Risk factors were explored separately by sex across these approximate NT-proBNP quintiles. In a further analysis, the patterns of sociodemographic and cardiovascular risk factors were explored specifically in young males and females (<40 years) by status according to whether or not NT-proBNP was moderately elevated at the ≥125 pg/mL threshold. For these analyses, continuous risk factors were expressed as mean and SD if normally distributed, and median and interquartile interval if skewed. Categorical risk factors were expressed as number and percentages.

Associations of elevated NT-proBNP with sociodemographic and classical cardiovascular disease risk factors were further investigated using logistic regression with robust standard errors. Missing data for classical risk factors were imputed by multiple chained imputations over ten datasets. In these models, the outcome was moderately elevated NT-proBNP ≥125 pg/mL. In a first regression model, age categories (by decade) and sex (with an interaction between age and sex) were used as exposure variables. In a second regression model, diabetes, current smoking, estimated glomerular filtration rate, blood pressure lowering medication use, SBP, BMI, cTnT, total cholesterol, and HDL-cholesterol were added, allowing for a sex interaction with each variable. Cholesterol-lowering medications were not added to the model, as there was no association with elevated NT-proBNP in either sex after adjustment. In an exploratory clustering analyses by family group, the intraclass correlation coefficient for NT-proBNP within families was 0.096 (95% CI, 0.082–0.111), indicating minimal family clustering; this was therefore not considered a factor in any other analyses.

All statistics were performed using STATA version 17.0.

## Results

### Population Characteristics

Of the 21 476 GS:SFHS participants 19 468 provided a serum sample with sufficient volume for measurement of NT-proBNP, as well as cTnI and cTnT (90.7%). After exclusion of 1110 participants with previous self-reported heart disease or stroke or previous hospitalization for coronary heart disease, heart failure, or ischemic stroke, and exclusion of 2 participants with no data on time of study appointment, there were 18 356 participants included in the study. Mean age was 46.1 years (SD 14.7 years), and 7503 participants (40.9%) were males. The median NT-proBNP was 50 pg/mL (interquartile interval 26, 90 pg/mL). Detectable (>10 pg/mL) levels of NT-proBNP were found in 17 071 participants (93.0%), and NT-proBNP was detectable in 97.8% of females and 86.0% of males.

### Diurnal Variation of NT-proBNP

**Figure 1.**
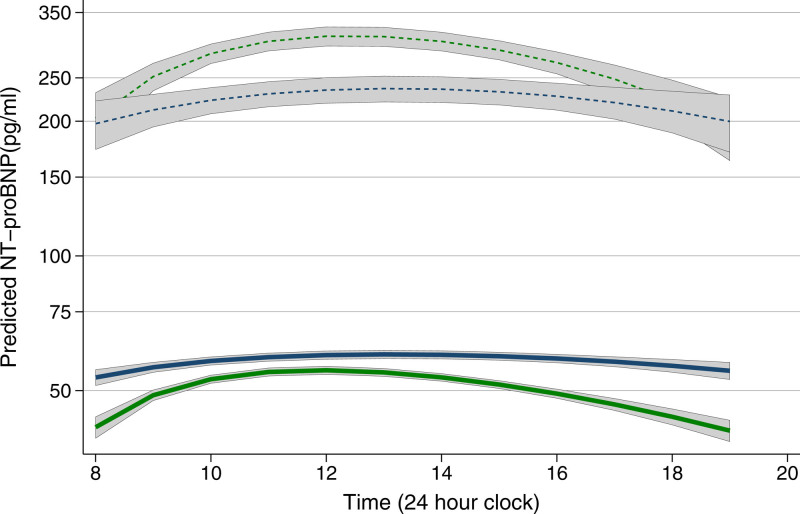
**Diurnal variation of NT-proBNP (N-terminal pro-B-type natriuretic peptide).** Association of the time of study assessment visit with NT-proBNP unadjusted (green) and adjusted for age and sex (blue). The solid line of each color represents the median, and the dotted line of each color represents the 97.5th centile. Clear areas with black outlines represent 95% CIs for each colored line.

In an unadjusted model there was some apparent evidence of diurnal variation of NT-proBNP depending on the hour of the study assessment; however, this trend attenuated toward the null after adjustment for age and sex (Figure 1). This is primarily because participants attending appointments in the middle of the day tended to be in the older age categories (Table S1). Since subsequent models reported here are stratified by age and sex, or adjust for it, diurnal variation was not considered as a covariate in other analyses.

### Prevalence of Moderately Elevated NT-proBNP

**Figure 2.**
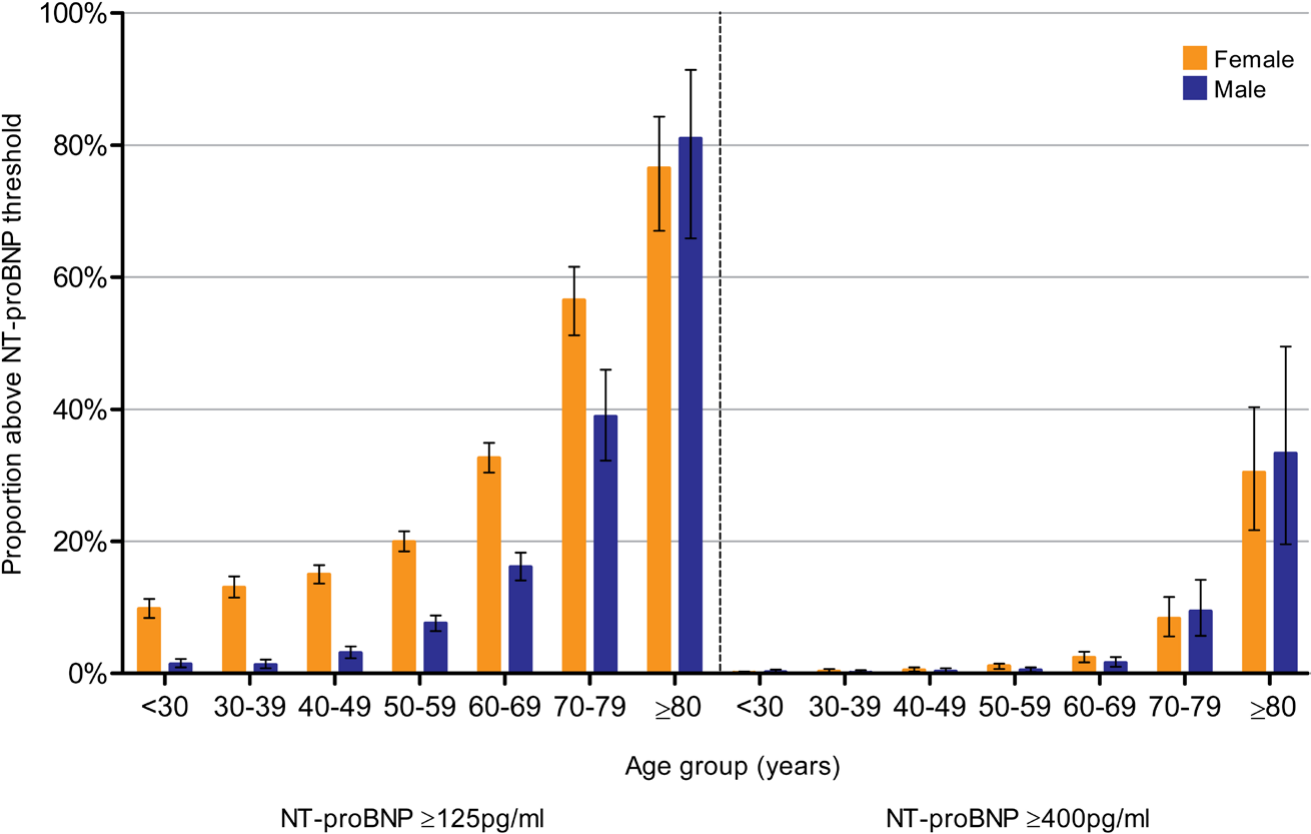
**Prevalence of moderately elevated NT-proBNP (N-terminal pro-B-type natriuretic peptide).** Proportion of participants (n=18 356) with moderately elevated NT-proBNP by age and sex.

Moderately elevated NT-proBNP (≥125 pg/mL) was detected in 2686 participants overall, specifically in 19.8% of females and in 7.1% of males. At the age band of <30 years, 9.8% of females had NT-proBNP ≥125 pg/mL, rising to 76.5% at age ≥80 years (Figure 2; Table S2). Approximately 10% or more of females at every age strata had elevated NT-proBNP (Figure 2; Table S2). In males at age <30 years, 1.4% were above the ≥125 pg/mL threshold, rising to 7.6% at 50 to 59 years, and 81.0% at age ≥80 years.

Using the higher threshold of 400 pg/mL (advocated by National Institute for Health and Care Excellence as a rule-out threshold), <1% of males and females had NT-proBNP ≥400 pg/mL in age groups up to 40 to 49 years, and 30.4% of females and 33.3% of males had NT-proBNP above this threshold in the ≥80 years age category (Figure 2; Table S2).

### Reference Ranges for NT-proBNP

In males, median (97.5th centile) NT-proBNP concentration at age <30 years was 21 (104) pg/mL, rising to 38 (195) pg/mL at 50 to 59 years, and 281 (6792) pg/mL at ≥80 years. In females, median NT-proBNP at age <30 years was 51 (196) pg/mL, 66 (299) pg/mL at 50 to 59 years, and 240 (2704) pg/mL at ≥80 years (Table 1). Notably, median NT-proBNP was only higher in males than in females in the 80+ age category (Table 1). Males and females both generally had lower estimated glomerular filtration rate (eGFR) in the 80+ age category, which may partially explain some of the elevation in NT-proBNP in older participants (Table S3). However, reference ranges were broadly similar when restricting the cohort further to those with eGFR>60 mL/minute per 1.73 m^2^ (Table S4).

**Table 1.**
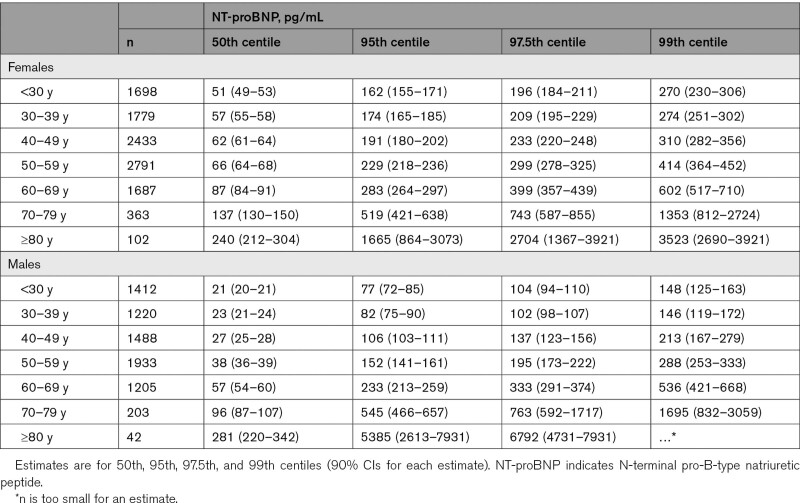
Reference Ranges for NT-proBNP Among 18 356 Participants With No Cardiovascular Disease

**Figure 3.**
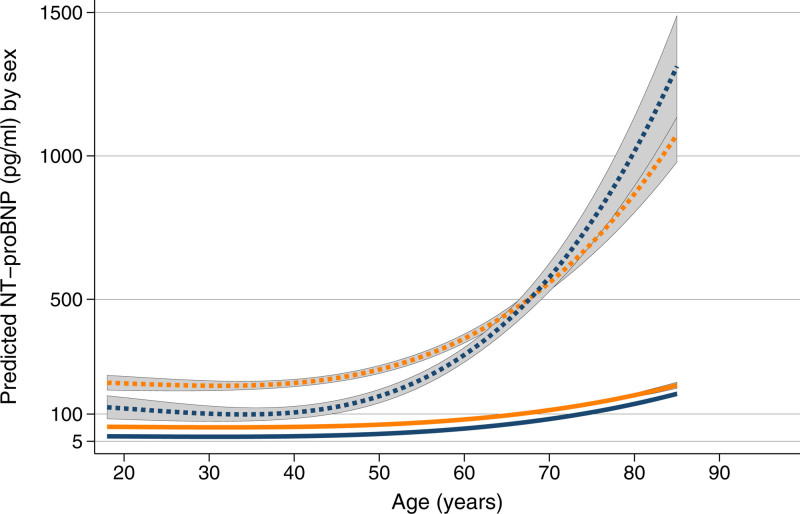
**Continuous model of expected NT-proBNP (N-terminal pro-B-type natriuretic peptide) by age.** Association between age and the 50th centile (solid line) and the 97.5th centile (dotted line) of NT-proBNP in females (orange) and males (blue) separately. Gray areas are 95% CI.

Continuous models of the median and 97.5th centile of NT-proBNP using quantile regression showed similar trends to the categorical age model, with generally higher levels of NT-proBNP in younger females, a slow rise in expected levels up to age 50 years, and then a more rapid rise in both sexes beyond the age of 50 (Figure 3). Predicted levels of NT-proBNP were generally higher in participants with poor renal function (Figure S1). Predicted levels of NT-proBNP were slightly higher in those with BMI <25 kg/m^2^, particularly among females (Figure S2).

### Sociodemographic and Cardiovascular Risk Factors Across Quintiles of NT-proBNP

Among female participants, those in the highest approximate quintile (≥125 pg/mL) for NT-proBNP were generally older, had higher SBP despite more frequently taking blood pressure lowering medication, had lower eGFR, higher cTnT and cTnI, and were more likely to be using cholesterol lowering medication. However, there was also a *U*-shaped association of NT-proBNP with diabetes and with BMI; females in the lowest NT-proBNP quintile were most likely to have diabetes and had the highest BMI. Females in the highest quintile also had slightly higher HDL-cholesterol and were less likely to be current smokers than females in the first quintile (Table 2). Patterns were similar in males, although males in the highest quintile had slightly higher BMI and were more likely to have diabetes than males in the lowest quintile (Table 3).

**Table 2.**
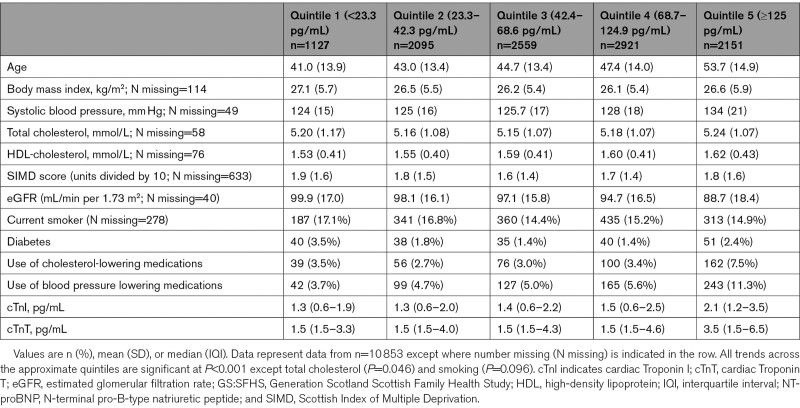
Population Characteristics in 10 853 Females in GS:SFHS, Stratified by NT-proBNP Across Approximate Quintiles, With the Upper Quintile Defined as Moderately Elevated NT-proBNP Above 125 pg/mL

**Table 3.**
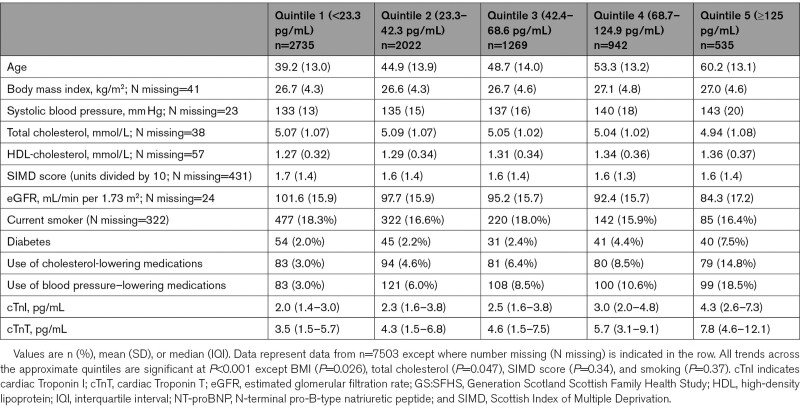
Population Characteristics in 7503 Males in GS:SFHS, Stratified by NT-proBNP Across Approximate Quintiles, With the Upper Quintile Defined As Elevated NT-proBNP Above 125 pg/mL

### Associations of Sex With Moderately Elevated NT-proBNP (≥125 pg/mL)

Using a model adjusting for age (and allowing for an interaction of age with sex), the odds ratio for moderately elevated NT-proBNP (≥125 pg/mL) was higher in females than in males (OR, 7.54 [95% CI, 4.72–12.1]). After adjusting for a panel of sociodemographic and cardiovascular risk factors (age, diabetes, SBP, total cholesterol, HDL-cholesterol, eGFR, smoking, blood pressure medication use, BMI and cTnT [plus allowing for sex interactions with each risk factor]) females still had higher odds of an elevated NT-proBNP (OR, 9.48 [95% CI, 5.60–16.1]).

Young males (<40 years) with moderately elevated NT-proBNP were more likely to be smokers and had higher cTnI and cTnT but also had lower BMI than young males who did not have moderately elevated NT-proBNP (Table 4). Among young females (<40 years), there was only a borderline trend that those with moderately elevated NT-proBNP had a slightly higher BMI. In general, in both sexes, young people with moderately elevated NT-proBNP had a generally low risk cardiovascular profile, including generally moderate cholesterol levels and blood pressure, good renal function, and low levels of cTnT and cTnI (Table 4).

**Table 4.**
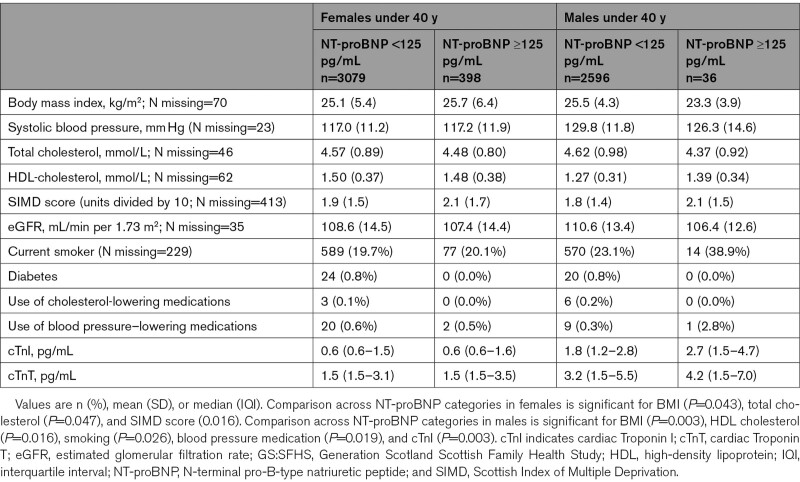
Population Characteristics in 3477 Females and 2632 Males Under the Age of 40 Years Separately, Stratified by Moderately Elevated NT-proBNP

### Associations of Age and Other Cardiovascular Risk Factors With Moderately Elevated NT-proBNP

**Figure 4.**
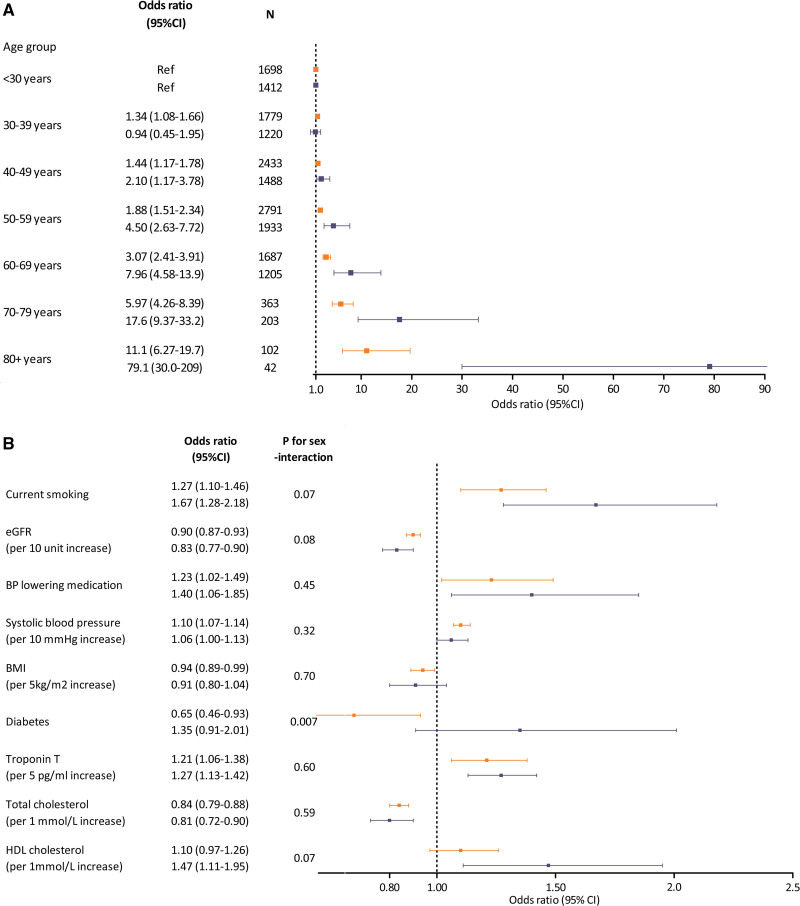
**Determinants of moderately elevated NT-proBNP (N-terminal pro-B-type natriuretic peptide).** Association of age (**A**) and other cardiovascular risk (**B**) with odds of moderately elevated NT-proBNP ≥125 pg/mL; females (orange) and males (blue). Squares are point estimates of the odds ratio, and horizontal lines are 95% CI. Each estimate is adjusted for age category, diabetes, current smoking, estimated glomerular filtration rate, blood pressure–lowering medication use, systolic blood pressure (SBP), body mass index (BMI), cTnT (cardiac Troponin T), total cholesterol and HDL (high-density lipoprotein)-cholesterol, and sex interactions for all variables. BP indicates blood pressure; and eGFR, estimated glomerular filtration rate.

Exploring different associations of cardiovascular risk factors with moderately elevated NT-proBNP in the sexes, age category was positively associated with moderately elevated NT-proBNP in both sexes after adjusting for other risk factors, although the association was stronger in males (*P*_interaction_ <0.001; Figure 4). Smoking was associated with increased odds of moderately elevated NT-proBNP in both sexes although the association was borderline stronger among males (*P*_sex interaction_=0.07). Use of blood pressure–lowering medication, SBP, and higher cTnT was associated with increased odds of moderately elevated NT-proBNP in both sexes to a similar extent. Higher eGFR was associated with lower odds of moderately elevated NT-proBNP in both sexes. Total cholesterol was inversely and HDL cholesterol positively associated with odds of moderately elevated NT-proBNP. BMI showed a borderline inverse association with moderately elevated NT-proBNP in both sexes. Diabetes was inversely associated with odds of elevated NT-proBNP in females but was not associated with odds of elevated NT-proBNP in males (*P*_sex interaction_=0.007). This divergence in the association of diabetes with NT-proBNP between the sexes was still observed in a sensitivity analysis when BMI, troponin, total cholesterol, and HDL cholesterol were eliminated from the adjustment model.

## Discussion

The recent universal definition of heart failure specifies an ambulatory NT-proBNP above 125 pg/mL as a corroborating factor when considering a diagnosis of heart failure when accompanied by symptoms or signs of the disease.^1^ Even outside of a diagnosis of heart failure or obvious structural heart disease, this threshold has also been linked to future cardiovascular events. Given the nonspecific nature of heart failure signs and symptoms, and prior data linking NT-proBNP values above this threshold with risk, results above this threshold might potentially be a cause for concern in many people and lead to further investigation. In this general population study, we report that females have a 7-fold increased odds of having an NT-proBNP above this threshold after adjusting for age. This observation directly contrasts with data suggesting the lifetime risk of developing heart failure is approximately equal between the sexes.^19^ Although many adverse cardiovascular risk factors were associated with higher odds of elevation in NT-proBNP, this did not explain these sex differences; even after adjusting for classical cardiovascular risk factors, females had 9-fold higher odds of NT-proBNP elevation. As such, these data suggest caution should be used in interpreting elevated levels of NT-proBNP above 125 pg/mL in the absence of corroborating evidence of heart failure, particularly in young females. These data are of relevance if general population NT-proBNP screening were to become more widespread.

NT-proBNP increases rapidly with age, and while this likely reflects greater underlying heart disease burden and lower eGFR in older people, there needs to be rationalization of what is normal in older age groups. The average age of diagnosis of heart failure in the United Kingdom is 77 years, and data from The Health Improvement Network (THIN) in primary care in Scotland suggests diagnosed heart failure prevalence in Scotland is 2.5% in 65 to 74 year olds and 6.3% in those aged over 75.^20^ The prevalence of undiagnosed heart failure in general population may be considerable.^21^ A meta-analysis based on echocardiographic screening studies in the general population, identifying undiagnosed heart failure, estimated that the prevalence of heart failure in developed countries is around 11.8% in those aged 65 years and over.^22^ Given the high prevalence of an “elevated” NT-proBNP in these age groups, use of a threshold of 125 pg/mL to determine the need for further investigation is likely to lead to many unnecessary echocardiograms in the diagnostic work-up of people with breathlessness and to make screening for undiagnosed cardiac dysfunction highly inefficient. As previously suggested by Hildebrandt et al,^14^ our data potentially support the use of age and sex-specific thresholds for NT-proBNP, and the threshold of 450 pg/mL is used in the United States for people over the age of 75 years. Further work is required to define clinically actionable thresholds in the general population.

It has been previously reported that young females have higher NT-proBNP than young males.^23^ Smaller studies have also reported reference ranges for NT-proBNP in broadly healthy populations, including the Gutenberg Health Study of 4266 subjects from the general population, and a study of 2812 patients attending a cardiovascular health screening programme in Taipei, Taiwan.^24,25^ Consistent with data in GS:SFHS, those studies also report higher levels of NT-proBNP in young females compared with males, and a strong trend across age ranges up to the age of 70 years. An individual participant meta-analysis of NT-proBNP also reported similar trends in graphical form, with median levels of NT-proBNP around 60 pg/mL in females and 30 pg/mL in males aged 40 years, rising to around 200 pg/mL in both sexes at age 80 years.^8^ Data from GS:SFHS expand on existing data importantly by providing clear reference ranges by decade of age and sex in a single large general population study.

We report an inverse association of NT-proBNP with diabetes in females; females with diabetes were less likely to have NT-proBNP above the 125 pg/mL threshold. In line with this, previous work has reported an inverse association between NT-proBNP and incident diabetes risk in females, but not in males,^26^ an observation supported by work in other cohorts.^27^ Mechanistically, the active BNP hormone stimulates lipolysis and may play a role in glucose homeostasis; transgenic mice overexpressing BNP are protected from diet-induced insulin resistance and obesity.^28^ Data from Mendelian randomization studies suggest that moderate lifelong elevations of BNP protect against type 2 diabetes.^29^ The existing literature therefore provides some validation and explanation of our finding that diabetes was associated with lower NT-proBNP in general in this broadly healthy cohort. Our observation that this seems particularly true in females has been previously reported^26^ but requires further work to explain the observation mechanistically.

The findings with respect to diurnal variation are of note; there is a mixed literature supporting and refuting diurnal variation in NT-proBNP.^30–33^ In a crude analysis, the apparent diurnal variation of NT-proBNP in the study was quite strong, but this trend attenuated substantially to the null when adjusting for age and sex.

Strengths of this study include the use of a general population, as well as the large size and the wide age range. NT-proBNP was measured using automated gold standard assays available to clinical biochemistry departments. Weaknesses include that the study does not have detailed echocardiographic or cardiac magnetic resonance imaging to exclude structural heart disease (even modest) in those with higher NT-proBNP concentrations. As such, given numerous studies linking modest NT-proBNP elevations with risk of heart disease, we cannot definitely state that the higher values seen in some individuals were normative findings. Additional limitations include that this cohort is not demographically representative of any national population, and is predominantly White,^15^ meaning generalizability to other races is limited.^18^ Some strata, particularly over 80s, have relatively few participants in the study, and the reference estimates have wide CIs. Previous heart failure was identified from routinely collected hospital data using ICD codes, meaning some misclassification is likely, and we cannot exclude participants with atrial fibrillation. For these reasons, this study has not sought to propose new reference ranges for heart failure rule-in or rule-out. Age of menopause or stage of menstrual cycle was not explored as exposures of interest in this study. The cross-sectional design of the study means causal inferences from associations should be made with caution, and regression models may be prone to unrecognized collider bias.

In conclusion, an NT-proBNP ≥125 pg/mL is observed in around 10% of young females and many do not have marked classical cardiovascular risk factors. A better understanding regarding determinants and ramifications of moderately elevated NT-proBNP concentrations are needed.

## Article Information

### Acknowledgments

The authors thank Philip Stewart, Elaine Butler, Emma Dunning‚ and Josephine Cooney (University of Glasgow, United Kingdom) for excellent technical support and Liz Coyle, University of Glasgow, for her assistance with manuscript preparation. The authors are grateful to all the families who took part, the general practitioners and the Scottish School of Primary Care for their help in recruiting them, and the whole Generation Scotland team, which includes interviewers, computer and laboratory technicians, clerical workers, research scientists, volunteers, managers, receptionists, health care assistants, and nurses.

### Sources of Funding

Roche Diagnostics supported this study through provision of free reagents and a grant. Generation Scotland received support from the Chief Scientist Office of the Scottish Government Health Directorates (CZD/16/6) and the Scottish Funding Council (HR03006). Dr Mills is supported by a British Heart Foundation Senior Clinical Research Fellowship (FS/16/14/32023). Dr Januzzi is supported in part by the Hutter Family Professorship Chair. Drs McMurray and Sattar are supported by British Heart Foundation Centre of Research Excellence Grant RE/18/6/34217. D.M. Kimenai was supported by Health Data Research UK, which receives its funding from HDR UK Ltd (HDR-5012) funded by the UK Medical Research Council, Engineering and Physical Sciences Research Council, Economic and Social Research Council, Department of Health and Social Care (England), Chief Scientist Office of the Scottish Government Health and Social Care Directorates, Health and Social Care Research and Development Division (Welsh Government), Public Health Agency (Northern Ireland), British Heart Foundation‚ and the Wellcome Trust. The funders had no role in the design and conduct of the study, in the collection, analysis, and interpretation of the data, and in the preparation, review, or approval of the article.

### Disclosures

Dr Welsh reports grant income from Roche Diagnostics within this work and grant income from AstraZeneca, Boehringer Ingelheim, and Novartis, and speaker fees from Novo Nordisk, outside the submitted work. Dr Sattar has received grant and personal fees from AstraZeneca, Boehringer Ingelheim, and Novartis; grant from Roche Diagnostics; and personal fees from Abbott Laboratories‚ Afimmune, Amgen, Eli Lilly, Hanmi Pharmaceuticals, Merck Sharp & Dohme, Novo Nordisk, Pfizer, and Sanofi outside the submitted work. Dr Januzzi is a Trustee of the American College of Cardiology; is a board member of Imbria Pharmaceuticals; has received grant support from Abbott Diagnostics, Applied Therapeutics, Innolife, and Novartis; has received consulting income from Abbott Diagnostics, Boehringer Ingelheim, Janssen, Novartis, and Roche Diagnostics; and participates in clinical end point committees/data safety monitoring boards for AbbVie, Siemens, Takeda, and Vifor. Dr McMurray reports payments to his employer, University of Glasgow, for work on clinical trials, consulting, lecturing, and other activities from Alnylam, Amgen, AstraZeneca, Bayer, Boehringer Ingelheim, Bristol Myers Squibb, Cardurion, Cytokinetics, Dal-Cor, GlaxoSmithKline, Ionis, KBP Biosciences, Novartis, Pfizer, and Theracos, as well as personal lecture fees from Abbott, Hikma, Sun Pharmaceuticals, and Servier. Dr Mills has received honoraria from Abbott Diagnostics, Siemens Healthineers, and Singulex, and the University of Edinburgh has received research grants from Abbott Diagnostics and Siemens Healthineers. All other authors declare no conflicts.

### Supplemental Material

Tables S1–S4

Figures S1 and S2

## Supplementary Material


