# Supplementary material for: Reference Ranges for NT-proBNP (N-Terminal Pro-B-Type Natriuretic Peptide) and Risk Factors for Higher NT-proBNP Concentrations in a Large General Population Cohort
Source: Circ Heart Fail. 2022 Sep 13;15(10):e009427. doi: 10.1161/CIRCHEARTFAILURE.121.009427 (PMC9561238; doi:10.1161/CIRCHEARTFAILURE.121.009427)

Supplemental Appendix

Reference ranges for NT-proBNP and risk factors for higher NT-proBNP concentrations in a large general population cohort

Welsh et al

Supplemental Table 1	page 2
Supplemental Table 2	page 3
Supplemental Table 3	page 5
Supplemental Table 4	page 8
Supplemental Figure 1	page 9
Supplemental Figure 2	page 10

Supplemental table 1 Attendance at the study assessment visit by age category

	Age category						
	18-29 years	30-39 years	40-49 years	50-59 years	60-69 years	70-79 years	80+ years
	N=3110	N=2999	N=3921	N=4724	N=2892	N=566	N=144
**Appointment time**							
7.00-11.00	1081 (34.8%)	1216 (40.5%)	1604 (40.9%)	1947 (41.2%)	1272 (44.0%)	238 (42.0%)	55 (38.2%)
12.00-16.00	1407 (45.2%)	1207 (40.2%)	1589 (40.5%)	1983 (42.0%)	1336 (46.2%)	294 (51.9%)	84 (58.3%)
17.00-20.00	622 (20.0%)	576 (19.2%)	728 (18.6%)	794 (16.8%)	284 (9.8%)	34 (6.0%)	5 (3.5%)

Chi squared test $p<0.001$

Supplemental Table 2 Proportion of participants (n=18,356) with elevated NT-proBNP above the Universal Definition of Heart Failure threshold (125pg/ml) and NICE rule-out threshold (400pg/ml), stratified by sex and age group.

	Female			Male		
**Category**	Percentage above threshold	Lower 95%CI	Upper 95%CI	Percentage above threshold	Lower 95%CI	Upper 95% CI
**ESC rule out threshold: 125pg/ml**						
**By age category**						
<30 years	9.8%	8.4%	11.3%	1.4%	0.9%	2.2%
30-39 years	13.0%	11.5%	14.7%	1.3%	0.8%	2.1%
40-49 years	15.0%	13.6%	16.4%	3.1%	2.3%	4.1%
50-59 years	19.9%	18.5%	21.5%	7.6%	6.4%	8.8%
60-69 years	32.6%	30.4%	34.9%	16.1%	14.1%	18.3%
70-79 years	56.5%	51.2%	61.6%	38.9%	32.2%	46.0%
80+ years	76.5%	67.0%	84.3%	81.0%	65.9%	91.4%
**By renal function**						
eGFR ≥90 mls/min/1.72m2	15.7%	14.9%	16.6%	4.4%	3.9%	5.0%
eGFR 60-89.9 mls/min/1.72m2	25.1%	23.7%	26.6%	11.3%	10.0%	12.6%
eGFR <60 mls/min/1.72m2	60.3%	53.7%	66.6%	47.5%	37.5%	57.7%
**NICE rule out threshold: 400pg/ml**						
**By age category**						
<30 years	0.1%	0.0%	0.3%	0.2%	0.0%	0.6%
30-39 years	0.3%	0.1%	0.7%	0.1%	0.0%	0.5%
40-49 years	0.5%	0.3%	0.9%	0.3%	0.1%	0.8%
50-59 years	1.1%	0.7%	1.5%	0.5%	0.2%	0.9%
60-69 years	2.4%	1.7%	3.3%	1.6%	1.0%	2.5%
70-79 years	8.3%	5.6%	11.6%	9.4%	5.7%	14.2%
80+ years	30.4%	21.7%	40.3%	33.3%	19.6%	49.5%

**By renal function**						
eGFR ≥90 mls/min/1.72m2	0.5%	0.3%	0.7%	0.4%	0.2%	0.6%
eGFR 60-89.9 mls/min/1.72m2	2.1%	1.6%	2.6%	1.5%	1.0%	2.1%
eGFR <60 mls/min/1.72m2	17.9%	13.3%	23.5%	15.8%	9.3%	24.4%

eGFR: estimated glomerular filtration rate; ESC: European Society of Cardiology; NICE: National Institute of Health and Care Excellence; NT-proBNP: N-terminal pro B-type natriuretic peptide

Supplemental Table 3 Population characteristics by age category in males and females in GS:SFHS.

	Age category						
	**18-29 years**	**30-39 years**	**40-49 years**	**50-59 years**	**60-69 years**	**70-79 years**	**80+ years**
**Females**	N=1698	N=1779	N=2433	N=2791	N=1687	N=363	N=102
Body mass index (kg/m^2) (N missing=114)	24.3 (5.1)	25.9 (5.8)	26.7 (5.7)	27.0 (5.5)	27.3 (5.3)	27.2 (4.9)	26.1 (4.5)
Systolic blood pressure (mmHg) (N missing=49)	116 (11)	118 (12)	124 (15)	133 (17)	140 (18)	148 (18)	155 (21)
Total cholesterol (mmol/L) (N missing=58)	4.39 (0.83)	4.73 (0.89)	5.13 (0.92)	5.64 (1.03)	5.72 (1.08)	5.44 (1.12)	5.38 (1.32)
HDL-cholesterol (mmol/L) (N missing=76)	1.50 (0.36)	1.51 (0.38)	1.57 (0.41)	1.65 (0.43)	1.65 (0.43)	1.65 (0.45)	1.65 (0.41)
SIMD score (units divided by 10) (N missing=633)	1.9 (1.5)	1.9 (1.5)	1.8 (1.5)	1.6 (1.4)	1.6 (1.4)	1.8 (1.6)	1.6 (1.2)
eGFR (ml/min/1.73 m2) (N missing=40)	112.8 (14.3)	104.3 (13.4)	95.8 (13.2)	89.4 (12.6)	83.6 (12.6)	73.1 (14.0)	63.2 (15.2)
Current smoker (N missing=278)	311 (18.9%)	355 (20.5%)	433 (18.2%)	333 (12.3%)	169 (10.2%)	30 (8.3%)	5 (5.0%)
Diabetes	11 (0.6%)	13 (0.7%)	35 (1.4%)	64 (2.3%)	48 (2.8%)	25 (6.9%)	8 (7.8%)
Use of cholesterol lowering medications	0 (0.0%)	3 (0.2%)	31 (1.3%)	132 (4.7%)	170 (10.1%)	78 (21.5%)	19 (18.6%)
Use of blood pressure lowering medications	3 (0.2%)	19 (1.1%)	61 (2.5%)	227 (8.1%)	247 (14.6%)	90 (24.8%)	29 (28.4%)

cTnI (pg/ml)	0.6 (0.6, 1.4)	0.6 (0.6, 1.6)	1.3 (0.6, 2.0)	1.8 (1.2, 2.6)	2.3 (1.6, 3.2)	3.3 (2.3, 4.7)	5.1 (3.4, 7.2)
cTnT (pg/ml)	1.5 (1.5, 3.0)	1.5 (1.5, 3.2)	1.5 (1.5, 3.6)	1.5 (1.5, 4.7)	4.3 (1.5, 6.7)	6.7 (4.6, 9.7)	10.4 (7.3, 14.1)
**Males**	N=1412	N=1220	N=1488	N=1933	N=1205	N=203	N=42
Body mass index (kg/m^2) (N missing=41)	24.4 (4.3)	26.6 (4.1)	27.4 (4.3)	27.7 (4.5)	27.4 (4.2)	26.8 (3.7)	25.4 (3.1)
Systolic blood pressure (mmHg) (N missing=23)	129 (12)	130 (12)	135 (14)	139 (16)	144 (18)	148 (18)	147 (17)
Total cholesterol (mmol/L) (N missing=38)	4.31 (0.88)	4.98 (0.98)	5.40 (0.98)	5.37 (1.02)	5.15 (1.03)	4.85 (1.01)	4.81 (0.99)
HDL-cholesterol (mmol/L) (N missing=57)	1.29 (0.30)	1.25 (0.31)	1.28 (0.33)	1.32 (0.35)	1.34 (0.37)	1.38 (0.39)	1.38 (0.37)
SIMD score (units divided by 10) (N missing=431)	1.7 (1.4)	1.8 (1.5)	1.7 (1.5)	1.5 (1.3)	1.3 (1.1)	1.4 (1.2)	1.8 (1.5)
eGFR (ml/min/1.73 m2) (N missing=24)	115.1 (12.8)	105.2 (12.1)	96.2 (12.8)	90.3 (11.5)	84.2 (11.8)	76.9 (13.2)	65.7 (15.4)
Current smoker (N missing=322)	307 (22.8%)	277 (23.9%)	265 (18.5%)	277 (15.0%)	106 (9.1%)	13 (6.6%)	1 (2.4%)
Diabetes	10 (0.7%)	10 (0.8%)	22 (1.5%)	77 (4.0%)	71 (5.9%)	19 (9.4%)	2 (4.8%)
Use of cholesterol lowering medications	0 (0.0%)	6 (0.5%)	36 (2.4%)	157 (8.1%)	172 (14.3%)	43 (21.2%)	3 (7.1%)
Use of blood pressure lowering medications	2 (0.1%)	8 (0.7%)	59 (4.0%)	179 (9.3%)	208 (17.3%)	49 (24.1%)	6 (14.3%)

cTnI (pg/ml)	1.6 (0.6, 2.6)	2.0 (1.4, 2.9)	2.3 (1.6, 3.6)	2.7 (1.9, 4.1)	3.2 (2.2, 4.8)	3.9 (2.9, 5.8)	6.8 (4.3, 14.2)
cTnT (pg/ml)	3.5 (1.5, 5.6)	3.1 (1.5, 5.3)	3.8 (1.5, 6.0)	4.6 (1.5, 7.2)	6.7 (4.5, 9.6)	9.9 (6.7, 13.4)	16.6 (12.0, 23.4)

Values are n (%), mean (sd), or median (IQI). Data represent data from n=7503 males and n=10,853 females except where number missing (N miss) is indicated in the row. All trends across age categories significant at p<0.001 in both sexes.

cTnI: cardiac Troponin I; cTnT: cardiac Troponin T; eGFR: estimated glomerular filtration rate; GS:SFHS: Generation Scotland Scottish Family Health Study; HDL: high-density lipoprotein; NT-proBNP: N-terminal pro B type natriuretic peptide; SIMD: Scottish Index of Multiple Deprivation

Supplemental Table 4 GS:SFHS reference ranges for NT-proBNP among 18,201 participants with eGFR ≥60 ml/min/1.73 m^2. Estimates are for 50th, 95th, 97.5th and 99th centiles, with 90% confidence intervals for each estimate.

		NT-proBNP (pg/ml)			
	n	50th centile	95th centile	97.5th centile	99th centile
**Females**					
<30 years	1,698	51 (49, 53)	162 (155, 171)	196 (184, 211)	270 (230, 306)
30-39 years	1,776	57 (55, 59)	174 (165, 183)	209 (195, 229)	274 (252, 316)
40-49 years	2,421	62 (61, 64)	190 (179, 202)	232 (220, 248)	307 (271, 330)
50-59 years	2,747	65 (63, 67)	225 (214, 233)	289 (272, 313)	389 (355, 438)
60-69 years	1,621	86 (82, 89)	273 (257, 291)	385 (347, 429)	586 (498, 697)
70-79 years	297	132 (122, 140)	424 (372, 527)	550 (468, 658)	688 (563, 830)
≥80 years	59	242 (212, 321)	1595 (708, 3133)	2364 (1367, 3133)	-
**Males**					
<30 years	1,412	21 (20, 21)	77 (72, 85)	104 (94, 110)	148 (125, 163)
30-39 years	1,219	23 (22, 24)	81 (75, 90)	102 (97, 107)	145 (120, 177)
40-49 years	1,483	26 (25, 28)	106 (101, 110)	135 (121, 155)	213 (165, 279)
50-59 years	1,919	38 (36, 39)	150 (141, 161)	191 (173, 224)	279 (249, 310)
60-69 years	1,160	56 (53, 59)	226 (207, 248)	319 (271, 367)	520 (391, 692)
70-79 years	180	93 (81, 101)	474 (380, 617)	798 (499, 1805)	1872 (676, 3059)
≥80 years	29	266 (200, 337)	5682 (2724, 7931)	-	-

-: indicates n is too small for an estimate; GS:SFHS: Generation Scotland Scottish Family Health Study; NT-proBNP: N-terminal pro B-type natriuretic peptide

Supplemental Figure 1 Association of age with median NT-proBNP (solid line) and 97.5th centile of NT-proBNP (dotted line), by sex separately, and stratified by eGFR categories (<70ml/min/1.73m²=red, 70-89.9ml/min/1.73m²=orange, ≥90ml/min/1.73m²=green). 95% CI not shown for clarity. eGFR: estimated glomerular filtration rate; NT-proBNP: N-terminal pro B-type natriuretic peptide

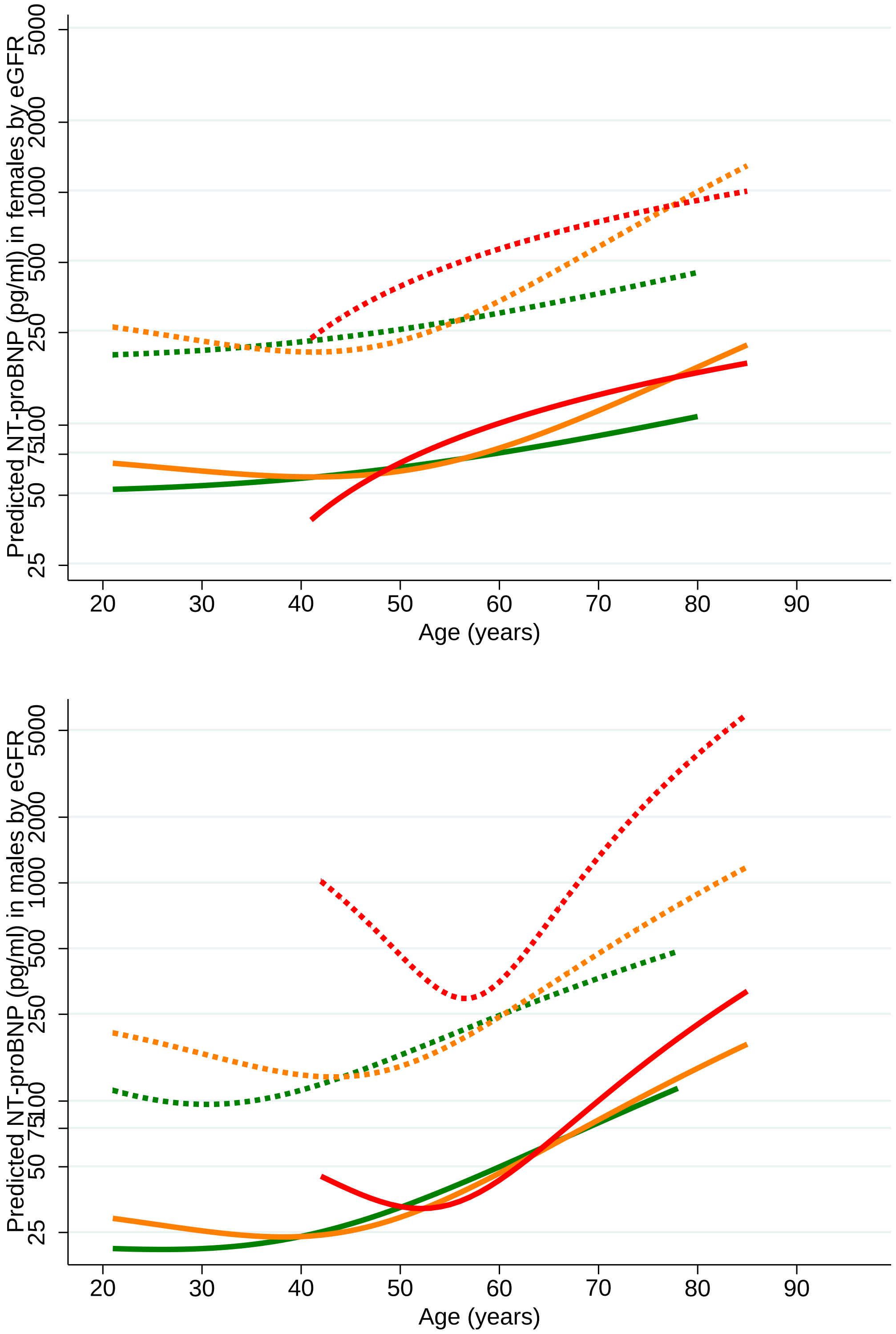

Supplemental Figure 2 Association of age with median NT-proBNP (solid line) and 97.5th centile of NT-proBNP (dotted line), by sex separately, and stratified by BMI categories (<25kg/m^2=green, 25-29.9kg/m^2=orange, ≥30kg/m^2=red). 95% CI not shown for clarity. BMI: Body mass index; NT-proBNP: N-terminal pro B-type natriuretic peptide

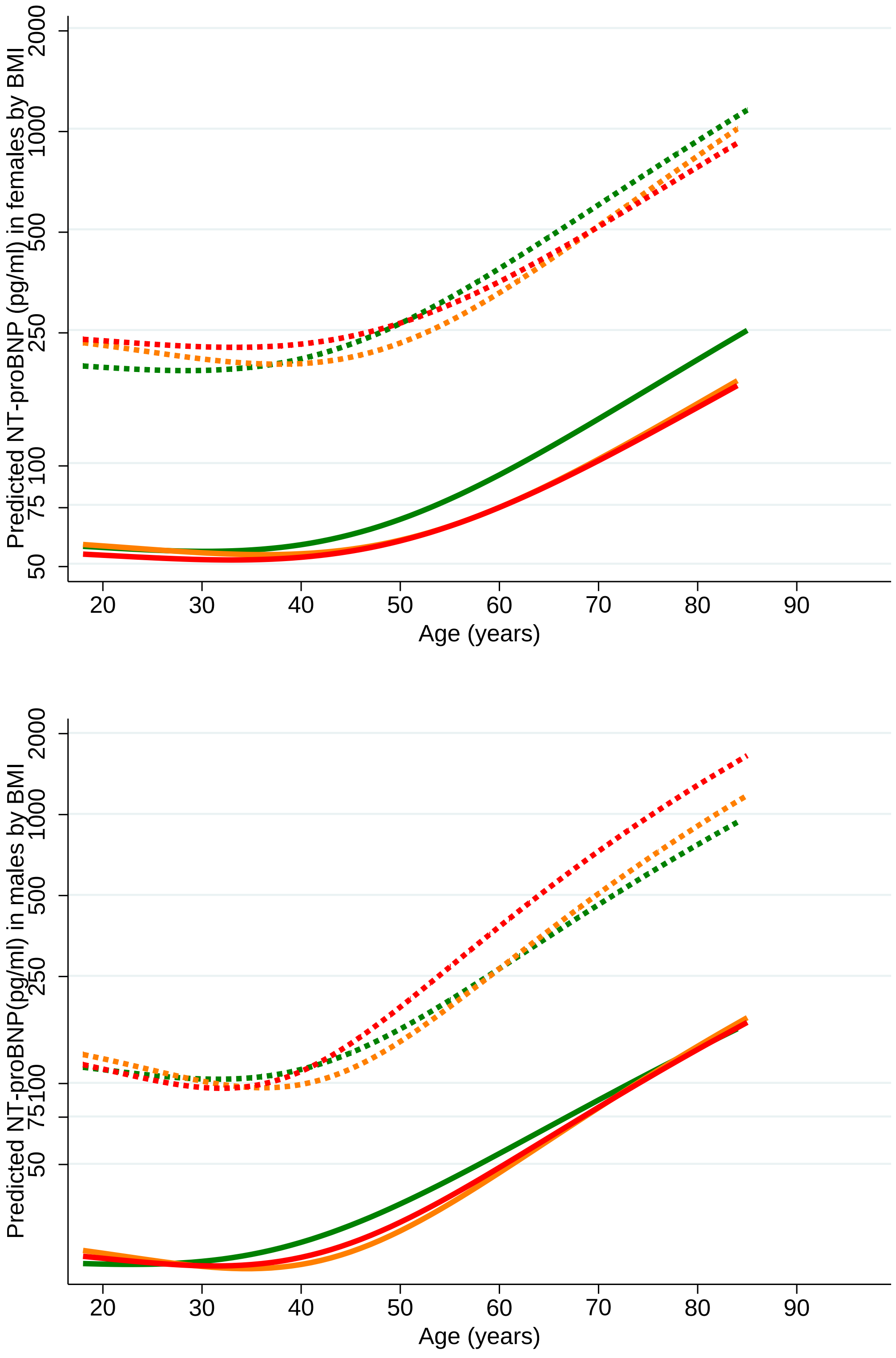

Supplement: Supplementary file 1 [file hhf-15-e009427-s001.pdf]
